# Supplementary material for: Implementing New Technologies to Improve Visual–Spatial Functions in Patients with Impaired Consciousness
Source: Int J Environ Res Public Health. 2022 Mar 5;19(5):3081. doi: 10.3390/ijerph19053081 (PMC8910167; doi:10.3390/ijerph19053081)
Supplement: Supplementary file 1 [file ijerph-19-03081-s001.zip › ijerph-1600507-supplementary.pdf]

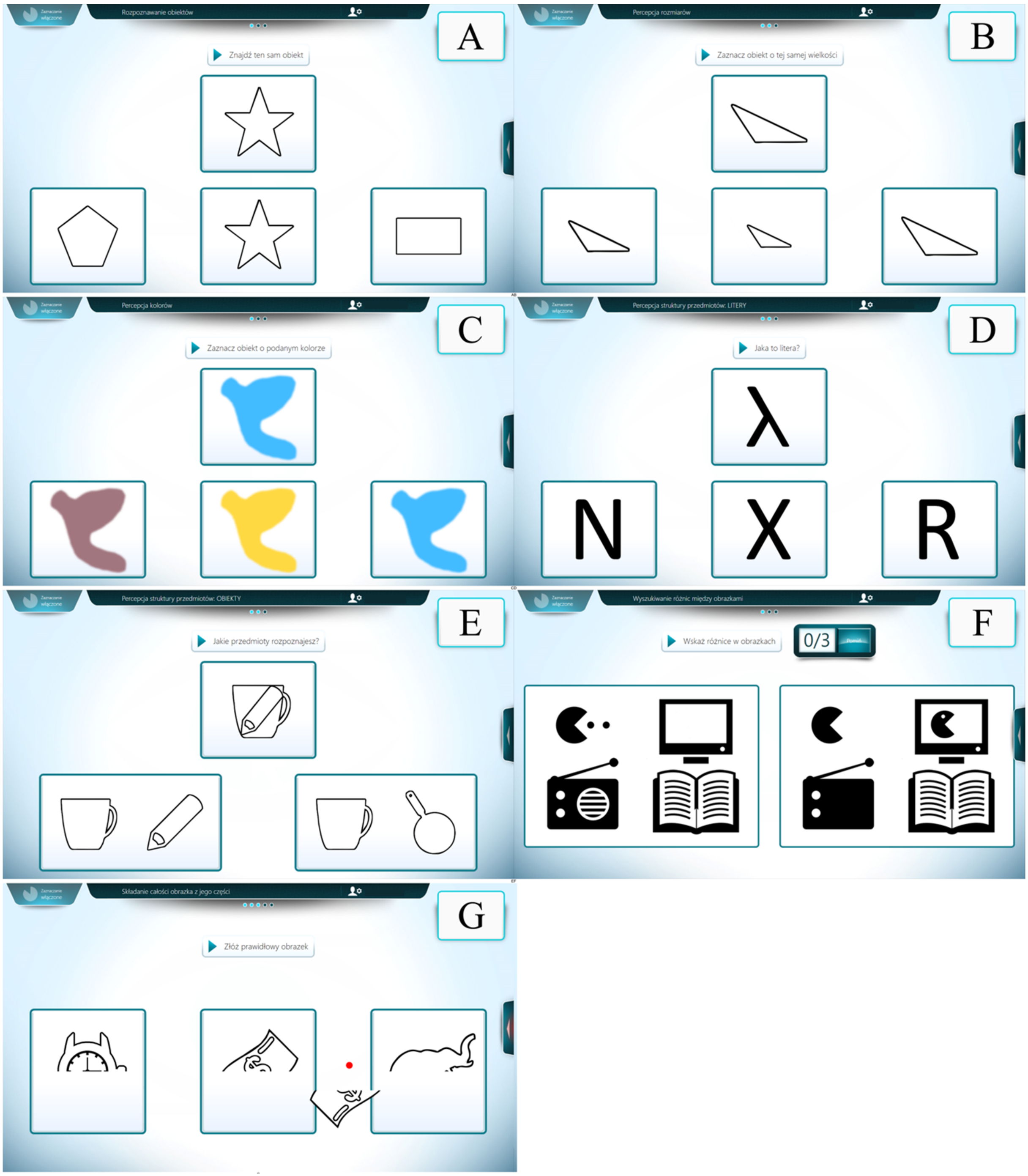

Figure S1. Categories of visual-spatial function testing: (A) recognising objects, (B) size perception, (C) colour perception, (D) perception of object structures – letters, (E) perception of object structures – objects, (F) finding differences between images and (G) assembling pieces of an image into the complete image.
